# Genetic rescue of absence seizures

**DOI:** 10.1111/cns.12858

**Published:** 2018-04-23

**Authors:** Cian McCafferty, William M. Connelly, Roberta Celli, Richard T. Ngomba, Ferdinando Nicoletti, Vincenzo Crunelli

**Affiliations:** ^1^ Neuroscience Division School of Bioscience Cardiff University Cardiff UK; ^2^ I.R.C.C.S Neuromed Pozzilli Italy; ^3^ Department of Physiology and Biochemistry University of Malta Msida Malta; ^4^ Present address: School of Pharmacy University of Lincoln Lincoln UK

- Crossbreeding GABA_A_R δ subunit knockout mice with stargazer mice removes the absence seizure phenotype
- Thalamic tonic GABA_A_ current is similarly abolished
- Rescue also occurs with acute siRNA knockdown of δ subunit
- Ataxic phenotype of stargazer is partially ameliorated

Absence seizures (ASs), the most common form of generalized epilepsy, have significant consequences for patients in the form of impaired attention, mood, and social deficits,^1^ and the potential for development into generalized tonic‐clonic seizures. Although mechanistic hypotheses of these paroxysmal oscillations are incomplete,^2^, ^3^ evidence suggests that an increase in extrasynaptic GABA_A_ receptor (eGABA_A_R) mediated tonic inhibition in thalamocortical (TC) neurons is sufficient to generate an AS phenotype in multiple rodent models.^4^ One of these models is the stargazer (STG) mouse, which has comorbid ataxia and features an early transposon insertion in the voltage‐dependent calcium channel (VDCC) subunit gene *Cacng2*, the protein product of which is known as stargazin. Loss of function in this mutated protein results in aberrant thalamic VDCC regulation and impaired cerebellar AMPA‐receptor trafficking, which have been implicated in the absence epileptic and ataxic phenotypes, respectively, of the STG mouse.^5^ In TC neurons, eGABA_A_Rs invariably contain a δ subunit, and tonic GABA_A_ current in these neurons has thus been shown to be dependent on the expression of that subunit.^6^ Further, knockout (KO) or suppression of the subunit (by RNA interference) reduces both TC tonic GABA_A_ current and the occurrence of absence seizures in the GHB (γ‐hydroxybutyrate) and GAERS (genetic absence epilepsy rats from Strasbourg) models of ASs, respectively.^4^ However, the role of the δ subunit and TC tonic GABA_A_ in the development of ASs remains to be demonstrated. Consequently, we investigated whether knockout of the δ subunit in STG mice, via crossbreeding them with δ subunit KO mice, could prevent the development of ASs, thus leading to a “genetic rescue” of the STG epilepsy phenotype.

Three‐month‐old F4 to F6 offspring from the breeding of B6C3Fe a/a‐Cacng2stg/J stargazer (STG) and B6.129‐Gabrdtm1Geh/J (δ KO) mice from the Jackson Laboratory were used for genetic rescue experiments. Stargazer mice from the same source were used for siRNA experiments. Data analysis and experimental procedures were similar to those previously described^4^, ^7^ and in accordance with the Animals (Scientific Procedures) Act 1986 (UK). Expression of the 2 transgenes (and GABA_A_R δ subunit) was investigated by PCR genotyping at postnatal day 21. ASs were detected from the presence of spike‐wave discharges (SWDs) on frontoparietal epidural EEG and of behavioral arrest on accompanying video recordings.^4^, ^8^ Tonic GABA_A_ current was measured in path‐clamped TC neurons of the ventrobasal thalamic nucleus in brain slices by adding the GABA_A_ blocker gabazine (100 μmol/L) in the presence of tetrodotoxin (1 μmol/L) and kynurenic acid (3 mmol/L) as previously described.^6^

**Figure 1:**
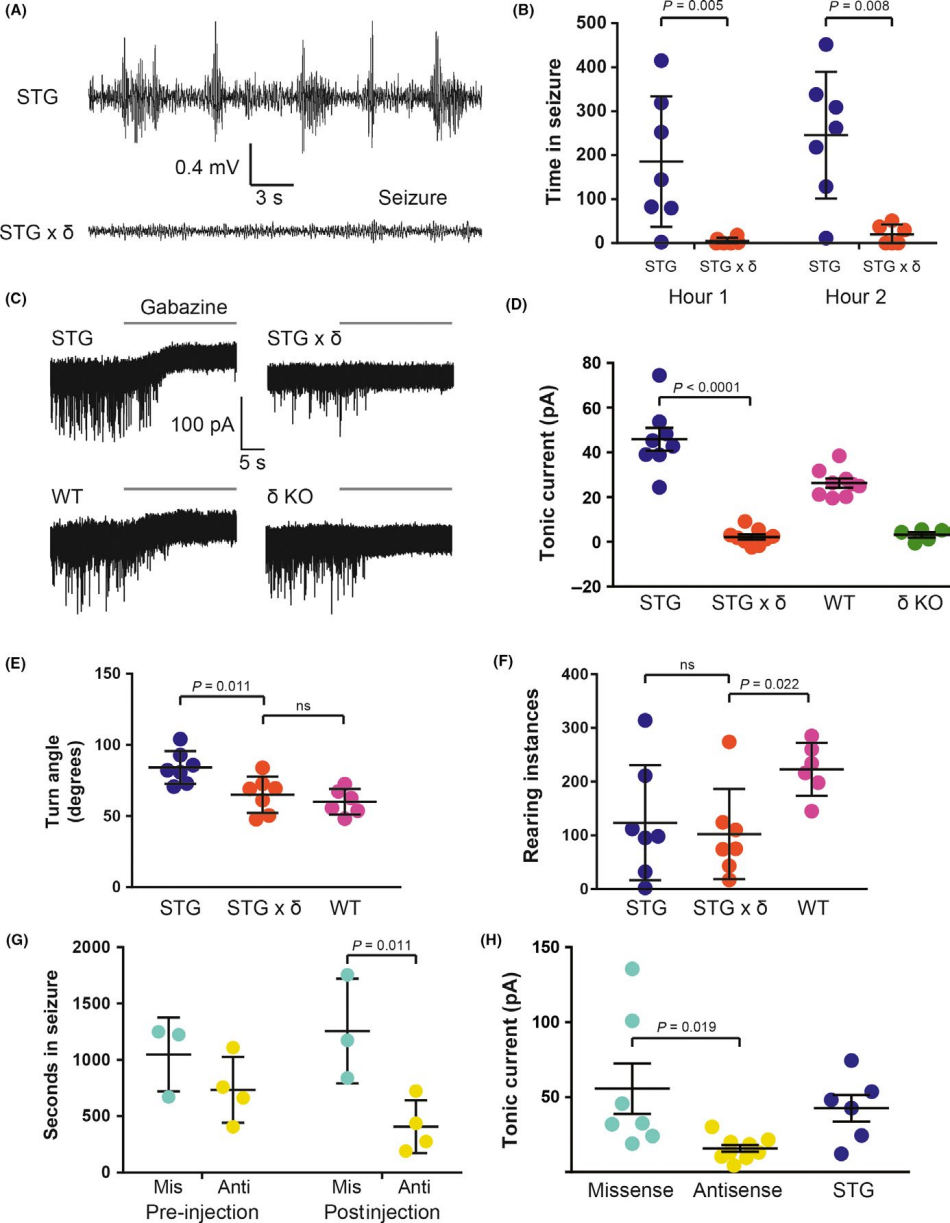
Genetic rescue of stargazer absence seizures by thalamic tonic GABA_A_ current abolition. A, Sample frontoparietal EEG traces from stargazer (STG) mice and STG mice crossed with δ subunit KO (STG × δ) mice, demonstrating spike‐wave discharges (SWDs, pale red areas) in the former and their absence in the latter. B, Total time spent in seizure in each of 2 recording hours for STG (blue, n = 7) and STG × δ (red, n = 6) mice demonstrating near‐complete abolition of ASs in the latter sample group (Mann‐Whitney, *P* < .01). In this and similar plots, horizontal lines indicate mean and SEM. C, Sample intracellular current traces from STG, STG × δ, wild‐type (WT, violet), and delta subunit knockout (δ KO, green), mouse thalamocortical (TC) neurons showing the effect of tonic GABA_A_ current block by administration of 100 μmol/L gabazine. WT and STG mouse neurons display gabazine‐induced outward current, indicating the existence of the tonic GABA_A_ current, whereas δ KO and STG × δ mouse neurons do not. D, Tonic GABA_A_ current amplitude (pA) in STG (n = 8), STG × δ (n = 9), WT (n = 9), and δ KO (n = 5) TC neurons illustrate elevated current in STG relative to WT and decreased current in both δ KO and STG × δ cells (*t* test, *P* < .0001). E, Mean turn angle through 1‐h open field session for STG (n = 7), STG × δ (n = 7), and WT (n = 6) mice, demonstrating larger turns in STG compared to both STG × δ and WT mice (Mann‐Whitney, *P* = .0156). F, Total instances of rearing throughout the same 1‐h open field session, showing similar counts for STG and STG × δ (Mann‐Whitney, *P* = .805), and a decrease in the latter group relative to WT. G, Time spent in seizure during predrug control and postinjection for STG mice with GABA_A_ δ subunit missense (cyan, n = 3) and antisense (yellow, n = 4) siRNA oligonucleotides injected into the ventrobasal thalamus, showing decreased time spent in seizure after antisense injection. H, Tonic GABA_A_ current in TC neurons from pairs of mice from the same oligonucleotide‐injected groups (n = 6 and n = 9 cells), and STG mice (n = 6 cells), demonstrating decreased tonic GABA_A_ current in the antisense‐injected mice compared to missense‐injected or naïve STG

We found that the STG × δ KO crosses (n = 6) averaged 94.3% fewer seizures (12.3 ± 6.1 compared to 215 ± 55.3 seconds per hour in seizure, *P* < .01, Mann‐Whitney) than STG (n = 7) across 2 hours (Figure 1A,B; Table 1) and that this suppression was accompanied by an absence of tonic GABA_A_ current in TC neurons (STG: 45.8 ± 5.1 pA, STG × δ KO: 2.1 ± 1.1 pA, *P* < .0001, Student's *t* test; Figure 1C,D). Some parameters of the ataxia phenotype expressed by the STG mice were also affected by the introduction of the δ KO. Thus, while the STG × δ cross was similar to wild‐type (WT) mice and improved from STG on measures (including turn angle, *P* = .011, Mann‐Whitney) relating to direction of movement (Figure 1E; Table 1), the effects on other locomotor measurements (including instances of rearing, *P* = .805, Mann‐Whitney) suggested limited or no recovery (Figure 1F; Table 1). We confirmed the attribution of the ASs suppression to the δ subunit KO by microinjection of short interfering RNA, antisense to the subunit, to the ventrobasal thalamic nucleus of 3‐month‐old STG mice. Mice injected with the antisense siRNA (n = 4) had 50.5% fewer seizures than control mice (n = 3) injected with missense RNA (570 ± 164 compared to 1152 ± 147 seconds in seizure over 2 hours, *P* = .011, *t* test). This was accompanied by a 71.5% decrease in TC neuron tonic GABA_A_ current in antisense (n = 10) vs missense‐injected (n = 7) animals (15.8 ± 2.2 compared to 55.7 ± 16.9 pA, *P* = .0185, *t* test).

**Table 1:** Properties of stargazer and stargazer × ∆KO seizures and locomotion

	STG		STG × δ		WT	
	Mean	SEM	Mean	SEM	Mean	SEM
Seizure						
Time in seizure (s)^a^	215.4	55.3	12.3	6.1		
Number of seizures^a^	82.2	22.5	6.2	3.7		
Seizure duration (s)^a^	8.5	2.3	2.5	1.4		
Movement						
Distance moved (cm)	5496	1084	8058	887	11789	1357.7
Velocity (cm/s)	4.6	0.9	6.7	0.7	9.8	1.1
Time moving (s)^a^	803.6	25.7	1027	19.5	942.8	57.1
Turn angle (°)^a^	84.2	3.9	64.9	4	60	3.7
Angular velocity (°/s)^a^	−106.5	12.3	−56.7	6.2	−47.1	6.1
Meander (°/cm)^a^	−52	4.6	−25.5	2.2	−26.3	4.7
Heading (°)	150.3	33	152.3	36.6	272.7	23.1
Rearing frequency	123.4	36.4	102.4	34.4	223	20.1

Mean and SEM values of seizure (per h) and locomotion properties for STG (n = 7) and STG × δ (n = 6) mice.

a Amelioration of seizure or ataxic characteristic in STG × δ mice (*P* < .05 in Mann‐Whitney test).

Our findings are in general agreement with the hypothesis that thalamic tonic GABA_A_ current has a crucial role in the mechanism of ASs in rodents. They also demonstrate the possibility of a “genetic rescue,” or prevention of seizure onset, by the abolition of tonic GABA_A_ inhibition in TC neurons throughout development. Consequently, the development of the AS phenotype from the stargazin mutation is entirely dependent upon the availability of the δ subunit, despite the direct role of the stargazin protein in VDCC regulation and AMPAR trafficking, 2 systems that may hitherto have been plausibly hypothesized to influence AS expression.^9^ By contrast, it is apparent that the ataxia also present in STG mice is not fully attributable to the δ subunit and may instead be related to the impairment of cerebellar AMPAR trafficking by the mutation of stargazin. However, our results also do not rule out a role for tonic GABA_A_ inhibition in the remaining locomotor phenotype due to the presence, in nonthalamic regions, of eGABA_A_Rs without the δ subunit.^10^

It is also noteworthy that, despite the elevated tonic GABA_A_ current in STG mice compared to WT, the current is completely abolished with the introduction of the δ KO. In other words, the mechanisms, downstream of the stargazin mutation, that increase tonic GABA_A_ inhibition in STG mice are completely dependent on the δ subunit and do not involve, for example, the insertion of other subunit configurations of eGABA_A_Rs. This is in stark contrast to the GABA_A_ receptor γ2R43Q mouse model, in which ASs are associated with a complete lack of thalamic tonic GABA_A_ current.^11^ The discrepancy may suggest that there exist multiple routes, including opposite disruptions of inhibitory balance, that result in ASs. This is supported by the existence of the same mutation in some human subjects with ASs.^12^ Moreover, the siRNA experiments demonstrate that the suppression of ASs by interference with the TC tonic current is effective whether delivered throughout development or acutely. Finally, the recent observation of elevated thalamic GABA in the thalamus of a child with absence epilepsy lends support to the potential translational application of this mechanism of rescue.^13^

In conclusion, this study is the first example of a genetic rescue of an absence seizure phenotype, confirming the centrality of TC neuron tonic GABA_A_ current in the mechanisms of rodent ASs, and therefore suggesting that therapeutic interventions for these seizures based on the disruption of this current, specifically via eGABA_A_ receptor containing the δ subunit, hold particular promise.
